# Correlation Between CT Severity Scoring and Diabetes Mellitus in Patients With COVID-19 Infection

**DOI:** 10.7759/cureus.20199

**Published:** 2021-12-06

**Authors:** Varsha Rangankar, Deepak V Koganti, Purnachandra Lamghare, Aparna Prabhu, Samanta Dhulipala, Parag Patil, Pratiksha Yadav

**Affiliations:** 1 Radiodiagnosis, Dr. D. Y. Patil Medical College, Hospital & Research Centre, Pune, IND; 2 Radiology, Dr. D. Y. Patil Medical College, Hospital & Research Centre, Pune, IND; 3 Medicine, Dr. D. Y. Patil Medical College, Hospital & Research Centre, Pune, IND

**Keywords:** severe, covid-19, hrct, diabetes, ctss

## Abstract

Background and objective

Severe acute respiratory syndrome coronavirus 2 (SARS-CoV-2), which causes coronavirus disease 2019 (COVID-19), was first identified in Wuhan, China in December 2019. Since then, It has spread across multiple countries and was declared a pandemic by WHO in March 2020. Patients with underlying diabetes mellitus (DM) are deemed at-risk for developing severe COVID-19 infection. In light of this, we aimed to evaluate the correlation between DM and chest CT severity scores (CTSS) in COVID-19 patients.

Methods

This was a hospital-based descriptive, analytical retrospective study conducted at our tertiary care hospital. A quantitative severity score was calculated among 220 patients with COVID-19 infection based on the degree of lung lobe involvement on CT chest scans. Based on CTSS, the patients were classified into groups of mild, moderate, and severe lung involvement. The association between DM and CTSS was evaluated using the chi-square test.

Results

The severity of lung involvement was higher among COVID-19 patients with a co-diagnosis of DM (29.3%) compared to those without DM (11.7%). This association of severe lung involvement with DM was statistically significant (p=0.002).

Conclusion

Based on our findings, diabetic patients are at an increased risk of developing the severe form of COVID-19 with a higher CT lung involvement score compared to non-diabetic patients.

## Introduction

In December 2019, the first case of coronavirus disease 2019 (COVID-19), caused by severe acute respiratory syndrome coronavirus 2 (SARS-CoV-2), was identified in the city of Wuhan in China. By January 2020, the virus had rapidly spread across the Wuhan province as well as other provinces in China [1]. It eventually spread across multiple countries and continents and was subsequently declared a pandemic by WHO in March 2020. The pathogen has been identified as a novel enveloped RNA beta-coronavirus [2].

Due to the disease's novelty, the criteria that influence the severity of the condition and mortality remain largely unclear. Patients with underlying health problems, people over 65 years of age, and delayed hospitalizations are all considered to play a role in the severity of the symptoms [3-5]. Patients with underlying medical disorders, including high blood pressure or diabetes, are deemed at-risk for developing severe coronavirus infection. Furthermore, such patients are thought to be more prone to developing subsequent complications, and their risk of dying from COVID-19 is high [3].

A worldwide prevalence of diabetes mellitus (DM) has affected approximately 463 million people [6]. Due to the vast prevalence of DM, India has been labeled the "Diabetes Capital of the World". Diabetes prevalence has been predicted to reach 11.3% among those aged between 20 and 79 years in the Southeast Asian region [7]. As of 2019, the total number of diabetes cases in Indian adults was approximately 77 million, and it is expected to reach 100 million by 2030 [8]. Numerous studies have shown that COVID-19 is more severe in patients with these comorbidities, with increased hospitalizations, ICU admissions, and ventilatory needs during the current COVID-19 pandemic [9]. A CT severity imaging score of >18 has been linked to a higher risk of death and has been proven to be predictive of death among patients [10].

The goal of this research was to fill the gaps in the existing literature by conducting a study that analyzed current data to determine the extent of the association between COVID-19 severity and DM. We hoped that our findings would contribute toward complication prevention and better patient management stratification.

## Materials and methods

Study design

We conducted a hospital-based descriptive and analytical retrospective study at our tertiary care hospital after obtaining approval from the Institutional Ethics Sub-Committee (IESC) of Dr. D. Y. Patil Medical College, Hospital & Research Centre, Pune, IND. The approval number assigned to our study is IESC/FP/2021/53.

Inclusion criteria

Patients of both genders and all age groups who had undergone high-resolution CT (HRCT) of the lung after testing positive for COVID-19 infection on reverse transcription-polymerase chain reaction (RT-PCR) were included in our study.

Exclusion criteria

Patients whose CT could not be evaluated due to severe motion artifacts or other technical issues such as restricted field of view or those with CT studies requiring contrast medium were excluded from our study.

Data collection

**Figure 1:**
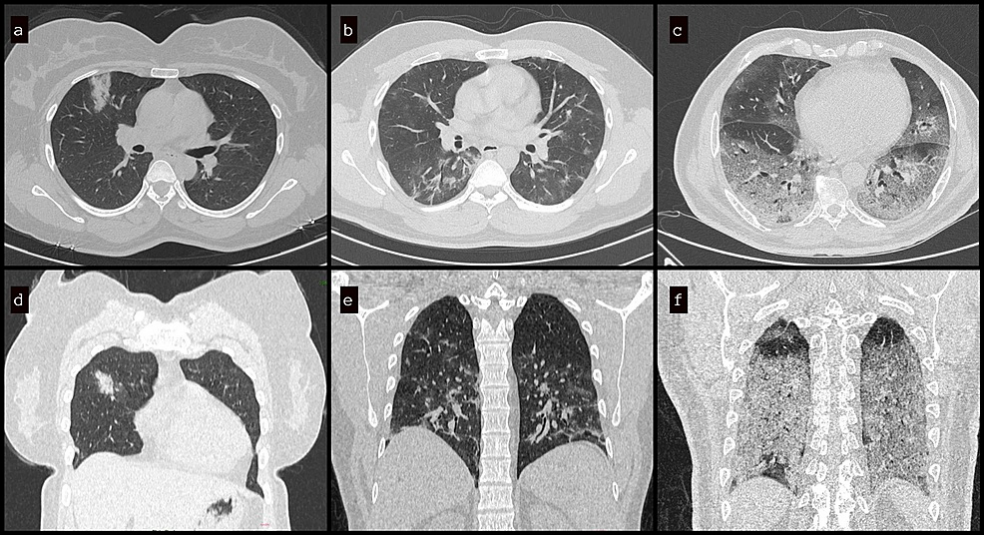
HRCT lung showing the extent of lung involvement in mild (a, d), moderate (b, e), and severe (c, f) disease based on CT scoring system HRCT: high-resolution computed tomography

The results of RT-PCR were considered confirmatory for the diagnosis of COVID-19 infection. The HRCT scans were performed between the fifth and 10th days after the onset of the symptoms. HRCT of the lung was performed on a multidetector 128-slice CT scanner (Philips Ingenuity Core, Philips Healthcare, Amsterdam, Netherlands). Patients were placed in the supine position and asked to hold their breath at the end inspiration for non-contrast-enhanced HRCT scans. The following technical parameters were used: tube voltage of 120 kV, tube current of 100-200 mAs, collimation of 1.5-3 mm, and slice thickness of 0.625-1.25 mm. The high-spatial-frequency (bone) reconstruction algorithm was used to evaluate the lungs. The data from the HRCT lung studies of 220 patients performed between March and May 2021, i.e., during the second COVID-19 wave, was retrospectively collected and analyzed to determine the association between diabetes and COVID-19 severity. All the scans were assessed by a senior radiologist with 15 years of work experience to avoid inter-observer variation in the severity scoring of the COVID-19 lung infection. A quantitative CT score was calculated based on the degree of lung lobe involvement (0: 0%; 1: <5%; 2: 5-25%; 3: 26-50%; 4: 51-75%; 5: >75%; range: 0-5 for each lobe; global score range: 0-25) [11]. Based on the CT severity score (CTSS), the patients were categorized into groups of mild (0-11/25), moderate (12-18/25), and severe (>18/25) lung involvement (Figure 1). The CTSS of the lung parenchyma involvement was compared between diabetic and non-diabetic patients. The analyzed data were used to validate the hypothesis that the severity of the lung involvement due to COVID-19 in diabetic patients is greater by using CTSS as evidence.

Statistical analysis

Statistical analysis was performed by entering the data into Microsoft Excel and evaluating it using the SPSS Statistics software version 17.0 (IBM, Armonk, NY). Patients’ gender, the status of DM, BMI, as well as ICU admissions were reported as percentages. The CT score was categorized into mild (0-11), moderate (12-18), and severe (>18) categories. Then mild and moderate groups were combined for the purpose of analysis. The association between DM and severe COVID-19 was calculated using the chi-squared test. A p-value <0.05 was considered statistically significant.

## Results

For the purpose of the present study, we retrospectively reviewed HRCT scans and the clinical data of 220 patients. Out of those 220 patients, 136 (61.8%) were males and 84 (38%) were females, with a mean age of 48.69 ±16.07 years (Table 1). No significant difference in terms of the course of the disease was observed based on gender. Normal (111/220, 50.5%) and obese categories (100/220, 45.5%) were almost equally represented in the cohort, and underweight patients accounted for 4% of the participants (Table 1). Out of 220 patients, DM was present in 41 (18.6%).

**Table 1:** Demographics and DM and ICU admission details of the RT-PCR‑confirmed COVID‑19 patients BMI: body mass index; COVID‑19: coronavirus disease 2019; DM: diabetes mellitus; RT-PCR: reverse transcription-polymerase chain reaction

Parameter	Total (n=220)	ICU admission not required (n=178, 80.1%)	ICU admission required (n=42, 19.1%)
Gender, n (%)			
Female	84 (38.2%)	67 (79.8%)	17 (20.2%)
Male	136 (61.8%)	111 (81.6%)	25 (18.4%)
BMI, n (%)			
Underweight	9 (4.1%)	8 (88.9%)	1 (11.1%)
Normal	106 (50.5%)	93 (87.8%)	13 (12.2%)
Overweight	105 (45.5%)	77 (73.2%)	28 (26.8%)
DM, n (%)			
Present	41 (18.6%)	25 (60.9%)	16 (39.1%)
Absent	179 (81.4%)	153 (85.5%)	26 (14.5%)

**Figure 2:**
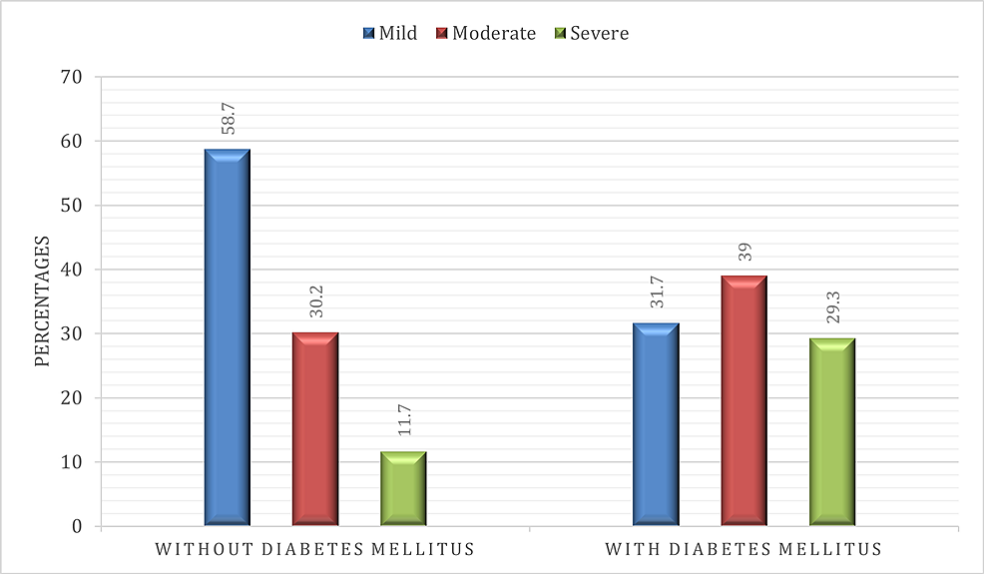
Bar chart showing the distribution of CTSS in patients without and with DM CTSS: chest computed tomography severity score; DM: diabetes mellitus

Out of 220 patients with COVID-19, more than half (118, 53.5%) had a milder form of lung involvement as per the CTSS category; 70 patients (31.8%) had a score of 12-18, which corresponded to moderate involvement, and the remaining 32 (14.6%) patients had a severe form of lung involvement (Table 2). Out of 179 patients without diabetes, 105 (58.7%) were affected by a milder form of the disease, 54 (30.2%) had moderate disease, and 20 (11.7%) had a severe form of lung involvement as per CTSS scores (Table 2). In patients with diabetes, 16 (39%) had a moderate form and 12 (29.3%) had a severe form of the disease. The proportion of patients with a severe form of COVID-19 lung involvement was higher in patients with DM (29.3%) compared to patients without DM (11.7%) (Figure 2). This association of severe lung involvement in COVID-19 infection with DM was statistically significant (p=0.002).

**Table 2:** Distribution of CTSS in patients with and without DM Chi-square p-value=0.002 (significant) CTSS: chest computed tomography severity score; DM: diabetes mellitus

Chest CTSS categories	Patients without DM, n (%)	Patients with DM, n (%)	Total, n (%)
0-11 (mild)	105 (58.7%)	13 (31.7%)	118 (53.6%)
12-18 (moderate)	54 (30.2%)	16 (39.0%)	70 (31.8%)
>18 (severe)	20 (11.7%)	12 (29.3%)	32 (14.6%)
Total (n=220)	179	41	220

## Discussion

According to existing research, diabetes appears to have a pertinent effect on clinical outcomes in COVID-19 patients [1,4,5]. As per most research, patients with diabetes who contract COVID-19 have a poorer prognosis and a higher mortality rate. The majority of the research on this topic has been done in China. According to a statement released by the International Diabetes Federation (IDF), symptoms among diabetes patients with COVID-19 are similar to those of other COVID-19 patients. Based on some reports, CT imaging findings differ across ICU patients, non-ICU patients, and recovered patients, implying that CT results can be used as an indicator for evaluating the severity of COVID-19 pneumonia [12]. Based on the measurable CT lung parenchymal involvement score, those with diabetes had a higher CT involvement than those without diabetes, indicating that diabetic patients had a more severe lung infection.

A meta-analysis including nine studies in China showed an association between diabetes and COVID-19 severity (OR: 2.67, p=0.01) [13]. The case fatality rate in diabetic patients was 7.3% in a study of 44,672 COVID-19 patients conducted by the Chinese CDC, compared to 2.3% in patients without diabetes [14]. SARS-CoV-2 has been shown to down-regulate the angiotensin-converting enzyme 2 (ACE2) protein [15], which might explain why COVID-19 patients with diabetes and cardiovascular disease had poorer clinical outcomes. In a study among Italian patients by Onder et al. [16], out of 355 patients who had died due to COVID-19, about 126 patients had diabetes (35.5%), which was the most common comorbidity attributed to COVID-19 deaths, followed by ischemic heart disease (30%). Only three patients (0.8%) had no comorbidities. According to a study by Wang G et al. [17], severe patients of COVID-19 had a higher incidence of diabetes (10.8% vs. 5.4%) than non-severe patients. Wang D et al. conducted a case series on 138 consecutive patients in Wuhan [18] and found that patients who required ICU treatment (n=36) were more likely to have diabetes than patients who did not require ICU care (n=102) (22.2% vs. 5.9%). Wu et al. conducted a study on 201 COVID-19 patients [19], among whom 84 patients (41.8%) had developed acute respiratory distress syndrome (ARDS). In patients with ARDS, there was a higher incidence of diabetes as compared to patients who did not develop ARDS. People with comorbidities like DM had a higher incidence of ARDS, which progressed to death.

It is unknown how diabetes worsens COVID-19, but numerous variables might be at play [20]. Inadequate viral clearance is caused by impaired T cell and NK cell function, as well as ineffective complement action. The incidence of infections of the lung due to viral and secondary bacterial etiology is increased due to glycaemic instability as it diminishes adaptive and innate immunity [21]. The proinflammatory state that already exists in COVID-19 might aggravate the cytokine storm. It is considered to be the cause of ARDS and systemic failure. In light of this, it is worth mentioning that type 2 diabetes and abnormal production of adipokines and cytokines have all been linked to weakened immunity and increased susceptibility to infection. Higher plasminogen levels have been associated with an increase in SARS-CoV-2 pathogenicity, which has been linked to diabetes [22]. The presence of these prothrombotic and inflammatory factors was discovered in a study on 174 COVID-19 patients in Wuhan, China, in which patients with diabetes had significantly higher serum levels of D-dimer, ferritin, erythrocyte sedimentation rate, C-reactive protein, interleukin-6, and fibrinogen [23]. Increased viral replication in diabetes might possibly be attributed to an increase in furin, a protease enzyme implicated in coronavirus entrance into cells [24]. Finally, hypoglycemia, which can occur with diabetes therapy, might exacerbate clinical results. Due to the ever-increasing number of diabetic patients and the high prevalence of COVID-19, it is clear that diabetic patient care must be improved in order to prevent future problems and mortality.

The relationship between COVID-19 and diabetes might be bidirectional, with SARS-CoV-2 possibly exacerbating underlying diabetes or perhaps predisposing non-diabetic individuals to diabetes. SARS-CoV-2 enters human cells through the ACE2 pathway, and ACE2 is widely expressed in the liver and endocrine pancreas, perhaps contributing to insulin resistance and reduced insulin production [25]. In susceptible individuals, infection of pancreatic beta cells may, in the long term, induce beta-cell autoimmunity. We can predict an increase in the incidence of autoimmune diabetes after the COVID-19 epidemic has ended [26]. While the immediate implications of the COVID-19 pandemic are an urgent concern that must be addressed as soon as feasible, the long-term effects of this virus should also be closely examined in the near future. Our study has some limitations, including the small sample size and limited laboratory data. Further studies involving a larger number of patients with intricate clinical and laboratory correlations will be helpful in drawing broader inferences.

## Conclusions

The present study has shown that diabetic patients are at an increased risk of developing the severe form of COVID-19 with a higher CT lung involvement score than non-diabetic patients. Thus, it is essential to ensure strict glucose monitoring in COVID-19 patients with DM to prevent the occurrence of life-threatening complications. Diabetes should be regarded as a risk factor for the severity of COVID-19 disease, and minimizing exposure to sources of COVID-19 is the best possible method to reduce morbidity and mortality.
